# A Survey of Methods and Technologies Used for Diagnosis of Scoliosis

**DOI:** 10.3390/s21248410

**Published:** 2021-12-16

**Authors:** Ilona Karpiel, Adam Ziębiński, Marek Kluszczyński, Daniel Feige

**Affiliations:** 1 Łukasiewicz Research Network—Institute of Medical Technology and Equipment, 118 Roosevelt, 41-800 Zabrze, Poland; daniel.feige@itam.lukasiewicz.gov.pl; 2 Department of Distributed Systems and Informatic Devices, Silesian University of Technology, 16 Akademicka, 44-100 Gliwice, Poland; Adam.Ziebinski@polsl.pl; 3 Department of Health Sciences, Jan Dlugosz University, 4/8 Waszyngtona, 42-200 Częstochowa, Poland; kluszcztroniny@gmail.com; 4 PhD School, Silesian University of Technology, 2A Akademicka, 44-100 Gliwice, Poland

**Keywords:** spine, diagnostic imaging, computer analysis, artificial intelligence diagnosis, scoliosis, spinal curvatures

## Abstract

The purpose of this article is to present diagnostic methods used in the diagnosis of scoliosis in the form of a brief review. This article aims to point out the advantages of select methods. This article focuses on general issues without elaborating on problems strictly related to physiotherapy and treatment methods, which may be the subject of further discussions. By outlining and categorizing each method, we summarize relevant publications that may not only help introduce other researchers to the field but also be a valuable source for studying existing methods, developing new ones or choosing evaluation strategies.

## 1. Introduction

**Figure 1:**
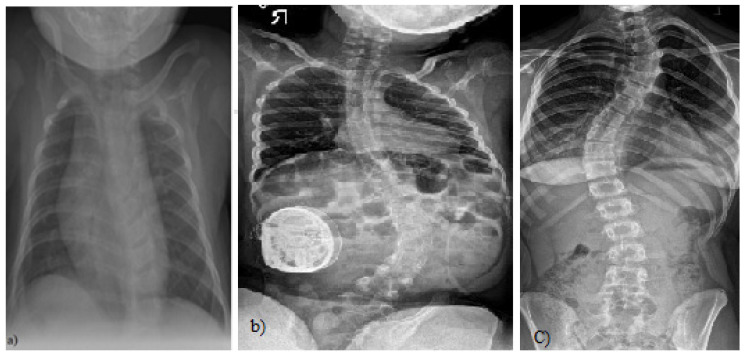
(**a**) A 4-month-old boy born small for gestational age, at 37 weeks, who presented initially with asymmetry of both the left and right aspects of the anterior and posterior chest and confirmed thoracolumbar scoliosis and vertebral anomalies based on plain radiography with a Cobb angle measurement of 30 degrees, (Case courtesy of Sonal Desai, Radiopedia.org, rID: 63310 under Creative Commons License (CC BY 3.0).) [5]; (**b**) X-rays of a girl with juvenile idiopathic scoliosis, (Case courtesy of Dr Jeremy Jones, Radiopaedia.org, rID: 89566 (CC BY 3.0).) [6]; and (**c**) severe left thoracic adolescent scoliosis, (Case courtesy of Dr Jeremy Jones, Radiopaedia.org, rID: 89456 (CC BY 3.0).) [7].

Scoliosis is defined as a three-dimensional spinal deformity consisting of a lateral curvature greater than 10 degrees with rotation of the vertebrae within the curve. It can be identified as congenital, neuromuscular or idiopathic. Idiopathic scoliosis (IS) can be further classified by age of onset: infantile (birth to two years), juvenile (three to nine years), and adolescent (10 years and older) (Figure 1). It is the most common pediatric musculoskeletal disorder that causes a three-dimensional (3D) spinal deformity [1]. The deformity is always 3D because it also involves an axial rotation of the vertebrae, not just displacement and rotation in the frontal plane. Adolescent IS is the most common form because the spinal deformity evolves during periods of significant physical growth [2]. IS is diagnosed when other etiological factors cannot be identified, such as congenital neurological or musculoskeletal anomalies, or inflammatory or demyelinating processes leading to primary or secondary motor neuron damage (myotonia, myopathy, etc.) [3,4].

The purpose of this research is to provide a brief review of diagnostic methods currently used in the diagnosis of scoliosis. This article aims to point out the advantages of select methods, which may be a valuable source of knowledge for future researchers. This article focuses on general issues without elaborating on problems strictly related to physiotherapy and treatment methods, which may be the subject of further discussions.

This paper is organized as follows: The first section (the Introduction section) provides a brief definition of scoliosis. The second section presents a brief historical background and traditional diagnostic methods. The third to sixth sections present methods of imaging and evaluation of scoliosis, including surface topography, raster topography, X-ray, magnetic resonance and computed tomography. The seventh section presents artificial intelligence methods for the detection of scoliosis. Section 8 describes open databases available for scoliosis detection. A short discussion of the methods presented is included in Section 9, and the conclusions are presented in Section 10.

## 2. Historical Background—Traditional Methods of Measuring the Degree of Spinal Curvature

Historically, scoliosis was analyzed using inclinometers, pantographs, and even plaster casts of the back [8]. Torsion-like growth changes occur both in the spine and throughout the trunk under the influence of modeling the loading and pulling forces by muscles and ligaments [9,10].

**Figure 2:**
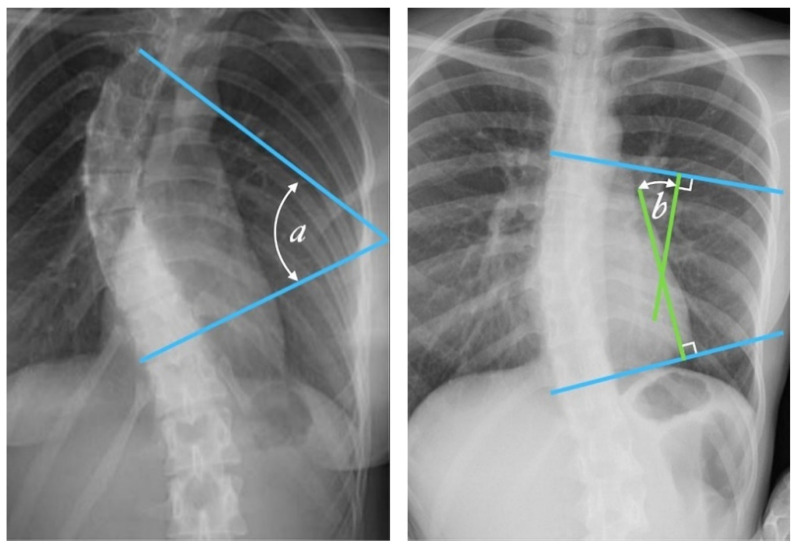
Cobb angle measurement. Tangential lines are drawn from the superior endplate of the superior vertebra and the inferior endplate of the inferior vertebra. The angle formed at the intersection of these two lines is the Cobb angle. A Cobb angle of at least 10 degrees is necessary for diagnosing scoliosis. (Case courtesy of Assoc Prof Frank Gaillard, Radiopaedia.org, rID: 49374, (CC BY 3.0).) [14] The Cobb angle is defined either as the angle between the tangential lines (angle a) or the angle between two lines drawn perpendicular (solid lines) to the tangents (angle b).

The primary diagnostic procedure used to monitor and assess the severity of scoliosis is spinal radiography. It is the gold standard for assessing spinal deformity but has negative long-term effects. One of the earliest methods was proposed in 1930 by Ferguson [11], who evaluated the deformity by determining the angle between the two straight lines that connect the centers of the end vertebrae with the center of the apical vertebra. Another similar method used to estimate the degree of scoliosis on a radiograph was proposed in 1948 by Cobb [12]. It consists of locating the most tilted vertebrae above and below the apex of the curve and measuring the angle between intersecting lines drawn perpendicular to the top edge of the top vertebrae and the bottom edge of the bottom vertebrae. The Cobb angle measurement has become the quantitative standard for recognizing and observing symptoms in scoliosis patients [13] (Figure 2).

Radiologists always measure the Cobb angle using a protractor after manually selecting the marginal vertebrae, which is presented in Figure 2. This angle is determined by drawing a line tangential at the superior endplate of the upper extremity curvature vertebra and at the inferior endplate of the lower extremity vertebra and then, lines perpendicular to each of the two lines at the most titled vertebrae [12,15]. The Cobb angle is useful in evaluating the initial curve, in determining the increasing magnitude of curves, and in deciding when surgical intervention may be most beneficial to the patient. The accuracy of the Cobb angle measurement mainly depends on the subjective experience of radiologists [13]. This method was used by many clinicians, and they presented the results based on the measurement error. According to some researchers, this error can be up to 11.8° [16]. Previously, measurements were made using a device called a Cobbometer, but the error was so large that it affected the diagnosis and treatment of patients with scoliosis. Thus, other methods of measuring the Cobb angle are developed to better assess the full three-dimensional spinal deformity with the modern imaging diagnostic techniques that allow 3D reconstructions [17]. Both the Cobb and Ferguson methods are based on manual identification of the end vertebrae. However, due to better reproducibility, easier application, and the ability to measure larger angles for more severe spinal curvatures, the Cobb method was preferred. The Cobb method has been standardized, and the key aspect of “reproducibility” has been tested and confirmed in numerous studies [18,19,20,21,22,23,24,25,26].

One of the simpler diagnostic procedures is the Adams Forward Bend test, which was described by William Adams in 1865 and allows for an assessment of posture and the possible determination of scoliosis. The Adams test belongs to the so-called functional tests. It is a short, non-invasive, painless test that does not require the use of any instruments and is often used by physiotherapists and orthopedists. During the test, the patient bends forward, and the physician stands behind their back and looks along the horizontal plane of the spine, seeking abnormalities of the spinal curve and estimating the angle of trunk rotation. Bunnel [27] proposed a simple handheld device called a scoliometer, which is often used to measure and evaluate the angle of trunk rotation during the Adams test and for screening purposes. In recent years, physicians have begun to notice that the detection of scoliosis based on in-office Adams tests may be insufficient. A negative result of this test usually causes the doctor to disregard a diagnosis of scoliosis because this test can only detect the disease when scoliosis is already quite advanced [28].

Surface deformation is recognized as not being able to accurately predict the severity of scoliosis, especially in younger children. Bunnell [29] stated that, although the correlation between clinical deformity and radiographic measurement is significant, the standard deviation is so large that reliably predicting the degree of curvature based on surface topography in a given patient is not possible using any technique. Still, in 2021, traditional screening for scoliosis is performed using the Adams test, and the Cobb angle measurement is considered the gold standard. Manual selection of vertebrae by clinicians consequently leads to inaccurate measurement accuracy. The result is affected by the choice of vertebrae and the bias of different observers. Nonetheless, these methods, in combination with modern imaging diagnostic techniques, can avoid or minimize the failures observed in the past.

Nowadays, the field of medicine is strongly associated with medical informatics. Almost every field of healthcare has been combined with different computer science techniques, giving great results, helpful in the evaluation of many conditions. Computer and software applications are great tools in the hands of physicians who know how to use them properly. As a result, medicine is becoming an interdisciplinary science. Many software applications are emerging to meet the needs of the medical community and many professionals.

## 3. Surface Topography Imaging and Spinal Deformity Assessment

Body surface topography (ST) is a photogrammetric technique; it deals with the reconstruction of shapes, sizes, and mutual positions of objects based on photogrammetric images (photograms). ST involves imaging and analyzing the external contours of the torso, usually from the backside of the subject. It has been successfully used to assess trunk deformities in children with scoliosis, where the relationship between the angle of spinal curvature and surface deformity is exploited. The undoubted advantages of ST include the non-invasiveness and safety of the examination, fast and accurate assessments of body posture in three planes of space, computer data storage and the acceptance of the examination by school-age children and adolescents.

### 3.1. Moiré Method

The Moiré pattern technique, sometimes called the projection Moiré technique, is a method of spatial photogrammetry (phototopography) that deals with the reconstruction of shapes and positions as well as the measurement of spatial objects based on so-called photograms, i.e., special photographic images. Moiré bars are a kind of arrangement of bars created as a result of interference (overlapping) of two grids of lines rotated by a certain angle or deformation (distorted with respect to each other). The development of the Moiré method occurred in two directions: The traditional raster was replaced by an optical one, which is a diapositive of stripes projected on the surface examined from a slide projector. The second direction is associated with the development of modern computer techniques.

The general principle of obtaining information concerning the shape of the surface with the use of the Moiré technique is based on an analysis of the image of a linear grid (raster) displaced by optical means onto the examined surface [30]. A patient with scoliosis has a characteristic difference between the contour lines of the two halves of the body. Currently, the aim is to simplify and automate measurement methods; hence, an optical raster (diapositive of stripes projected onto the body of the examined person) is used, and a computer analysis of the image is obtained [31].

### 3.2. Raster Topography with Automatic Image Analysis

The development of computer techniques contributed to the method of raster topography with automatic image analysis (also called raster stereography) [32]. The traditional raster was replaced by an optical one, which is a diapositive of stripes projected onto the examined surface from a slide projector. A special optical system with a camera captures the image and transmits it to a computer.

### 3.3. Diers Formetic II 4D Optoelectronic Method

The Diers formetic II 4D optoelectronic method is harmless to the patient because it does not use ionizing radiation. It provides rapid, non-contact and automatic (the instrument detects specific anatomical landmarks without the use of markers) measurement and analysis of the spine using a light-optical method. The instrument provides the possibility to accurately calculate the midline of the spine as well as the rotation curve and excludes the most common measurement error, the so-called human factor. It is an ideal alternative to invasive examinations also due to its availability, where it is perfectly applicable before and immediately after therapy to evaluate the effects [33,34,35]. The examination is characterized by high accuracy, and thanks to a direct view of the examined area, the doctor can analyze it in real time.

The device for three-dimensional examination consists of two main parts. The first is a digital video camera, and the second is a projector. The equipment emits measurement beams and directs them to the patient’s spine. The data collected in this way are immediately transferred to a computer, and a special program creates a digital map of the indicated body part. The obtained measurements can be used at a later stage to diagnose the problem and to indicate the appropriate course of action in case of ailments. The three-dimensional analysis of the spine can also be used for pregnant women.

## 4. Methods for Imaging and Evaluation of Scoliosis Using Radiography

Radiography, commonly called X-ray [36], is very important in imaging the spine. It provides a basic image, giving a general picture of the possible two projections (anterior-posterior (AP)/posterior-anterior (PA) and lateral (LAT)). At one time, X-rays of the spine were very commonly performed, but over time, efforts have been made to limit patient exposure to X-rays. Between 1935 and 1965, the incidence of breast cancer was almost doubled [37]. Today, radiation doses are lower, but the number of x-rays that must be taken of children during adolescence after/or during diagnosis is at least 12. Unfortunately, the risk of cancer due to cumulative X-ray dose is several times higher in children than in adults [38,39,40].

The development of technology and computerization allowed for the use of optoelectronic methods to localize the problem of posture and body statics. Unfortunately, the irreplaceable advantage of X-rays so far is the possibility of calculating the angle of torsion using the Cobb method and observing morphological changes in the vertebrae. As previously mentioned, an X-ray is an examination that carries harmful radiation, which means that the diagnosis is usually stretched over time. Medical personnel are not able to precisely determine whether the applied treatment process proceeds properly or whether it brings the desired results. Therefore, the ideal diagnostic tool is computer diagnostic methods; they are precise and non-invasive without the harmful effects of X-rays. Computer methods testing the posture are of practical importance because they allow us to catch the first signs of curvature. Additionally, they allow us to observe the patient’s body in all planes and to localize the problem, which may not yet be visible to the naked eye. Computerized methods of posture examination include the Moiré bar method, ISIS method, Posturomet-S, Metrecom System method and Diers formetric III 4D optoelectronic method [33,34,41,42,43,44,45,46].

## 5. Method for Imaging and Evaluation of Scoliosis Using Magnetic Resonance Imaging (MRI)

Magnetic resonance imaging (MRI) is a non-invasive method that is finding more and more applications, mainly in the development of specialized methods and sequences. The test uses a hydrogen atom, which makes the magnetic resonance process possible because it has a spin and a magnetic moment. The individual magnetic moments returned are disordered, but when a strong external magnetic field (B_0_) is applied, the magnetic moment returns are ordered—vectors parallel or anti-parallel to the main magnetic field. Atoms with an odd number of protons and/or neutrons can be visualized as spinning charged spheres with a small magnetic moment. An MR scanner has three magnetic fields that interact with these spinning spheres, commonly called spins, namely, the main magnetic field (B_0_), the radio frequency (RF) field (B_1_) and the gradient field (G). Under the external influence of a magnetic B_0_, some of the spins are aligned with it and hence have a net nonzero magnetic moment.

Following excitation by an RF pulse (B_1_), the net magnetization vector is tipped into the transverse plane, where it rotates about the external field at the Larmor frequency, giving rise to the MR signal. A second action of the RF pulse causes the spins to become aligned in orientation or to become phase coherent in the transverse plane. Over time, it recovers back to equilibrium, with the individual spins returning to their parallel or anti-parallel orientations and losing their phase coherence. As a result, it reforms along the z-axis, parallel with the applied main magnetic field, and with a magnitude of M0. This return to equilibrium is characterized by two orthogonal processes: longitudinal (T1) and transverse (T2) relaxation, governed by the T1 and T2 relaxation time constants. T1 relaxation describes the recovery along the longitudinal (z) direction (with the T1 being the time corresponding to the recovery of 63% of the equilibrium value), whilst T2 characterizes the loss of phase coherence in the transverse plane (with the T2 time corresponding to the loss of 63% of the initial value).

This signal is detected by specially designed RF coils and sent to a computer for image reconstruction. The times at which the excited atoms of the tissues under study return to equilibrium, or relaxation times, are represented by different shades of grey in the image [47].

This phenomenon is possible because hydrogen is part of the water molecule, which makes up 60–70% of the human body. Additionally, hydrogen is located in fat. The way hydrogen is distributed in different parts of the body is a parameter that differentiates different structures. Both the relaxation times and the density of protons affect the brightness, which is the degree of grey obtained in an image. The examination is associated with a strong magnetic field; for this reason, it is not recommended for patients with metal implants. The health risks resulting from the examination are very small, usually associated with the occurrence of allergic reactions immediately after the administration of the contrast medium.

The second stage involves detecting the MR signal and reconstructing it to create an image and is termed ‘acquisition’. Spatial encoding of the MR signal requires localization in three dimensions. In single-slice Cartesian 2D imaging, one first excites the nuclear spins in a thin slice, then plays a phase-encoding gradient pulse to impose a definite phase relationship across an in-slice direction, and finally reads out the signal, while a linear magnetic field gradient is played in the perpendicular in-slice direction (frequency encoding). This sequence of RF and gradient pulses is repeated for each phase encoding gradient, and finally, a 2D Fourier transform of the acquired signal reconstructs the image [48].

Most of the modern diagnostic methods today are widely used in many specialty fields. The fields of physics, computer science and medicine can be said to have been combined. MRI can be performed on virtually any part of the body using an appropriately selected sequence.

On the one hand, society is demanding greater accessibility for diagnostic support, particularly related to MRI access and scoliosis assessment. MRI is used in the diagnosis of patients with scoliosis primarily to evaluate neural structures and the shape of the spinal canal. Of note, this examination should not be repeated more than once. The routine, preoperative use of MRI remains controversial and current indications for MRI in idiopathic scoliosis vary from study to study (e.g., early scoliosis) [49]. The literature suggests and even excludes the use of MRI in specific cases such as routine preoperative MRI in idiopathic scoliosis unless the patient has neurological deficits [50,51]. MRI are used in the suspicion of congenital bone defects of the spine, e.g., Klippel-Feil syndrome, underdevelopment of the vertebrae, semivertebrae, intermolar adhesions, adhesions of articular processes, rib adhesions and bone blocks. Nerve bone defects, e.g., meningeal hernia (myelocele, myelomeningocele), were also observed. In addition, in the diagnosis of the nervous system, e.g., Recklinghausen’s disease, spinal tumors, syringomyelia, Arnold Charie’s syndrome. MRI is also indicated for scoliosis with an atypical pattern (for example, left thoracic scoliosis), in the diagnosis of congenital curvature of the spine and for concomitant neurologic disorders to detect nervous system defects [52]. Scoliosis also causes a number of dysfunctions in a person who is sick. In addition, diseases emerge from the formation of scoliosis, such as syringomyelia [53], vertebral segmentation anomaly, intramedullary spinal tumor [54] or Chiari malformation [55].

Magnetic resonance imaging may be beneficial for patients with presumed idiopathic scoliosis, and its non-invasiveness and precision contribute to improved diagnosis in the youngest patients without unnecessary exposure to X-rays.

Measurement methods have evolved sequentially since about 2002, where Rogers et al. [56] presented a method based on measuring intervertebral rotation in the lumbar spine. The method has found application in both MRI and CT [57].

Unfortunately, because MRI scans are expensive, they have been limited to studies of patients with congenital and severe curvatures [58]. Medicine of the 21st century is more and more personalized, where we observe the development of dedicated implants. A dedicated implant is a solution that is more and more often used in spine surgery when it is necessary to recreate the correct curvature of the spine, which has been lost as a result of degenerative disease, or as a result of a congenital defect or a complicated disorder of the spine axis. Such a spine is unable to maintain a proper line and tilts to the side or rotates or slides forward.

Materials from which the implants are made include polyetheretherketone (PEEK), titanium [59], cobalt-chromium [60], or other materials, e.g., bio-absorbable materials. The former is transparent to X-rays; therefore, these implants contain small radiographic markers. Titanium implants, on the other hand, are visible on radiographs and safe in MR imaging [61].

## 6. Computed Tomography (CT)

Although 2D images are still widely used in clinical research, advances in medicine have led to the development of a new 3D technique, which has become an important modern tool, obtained using computed tomography (CT) [62,63] and magnetic resonance imaging (MRI). These methods are certainly being developed at a very fast pace, and these methods are completely automated or semi-automated (requiring little intervention). Computed tomography was quickly appreciated because of the difficulty of evaluating X-ray images, which were usually taken in two projections. However, this did not give a complete picture of the problem, and curvature assessment was not problematic.

Computed tomography has been successfully used to take cross-sectional images of the body parts examined since 1973 (introducing tomographs to hospitals).

The 20th and 21st centuries tightened the procedures related to the use of X-rays, introducing even more restrictions related to the application of radiological protection to the patient. Due to the desire to limit radiation exposure, cross-sections are usually made at the level of the border vertebrae, the vertebral column, and the pelvis [64]. With the ever-increasing number of medical images, more and more methods that are fully automated or semi-automated, i.e., requiring minimal manual intervention, have appeared; however, they apply mainly to digital radiography X-rays. In contrast, in CT examinations, the clinician must set adequate parameters to better check the disease or the degree of scoliosis. The parameters should be optimized, and they require very good knowledge of the influence of parameters on the results. Thus, using specially developed methods for quantitative assessment of spinal curvatures that can improve medical diagnosis, treatment, and management of spinal disorders is necessary and will support the work of doctors.

Enhancing the CT method with three-dimensional image processing is possible. This allows for spatial imaging of the spine, the detection of spinal canal deformities, the detection of congenital malformations of the spine, the visualization of the location of spinal implants, and the assessment of the quality of spondylodesis. This examination plays an important role in the choice of surgical technique.

## 7. Artificial Intelligence (AI) As a Method for Detection of Scoliosis

Theories of artificial intelligence: neural networks mirror the behavior of the human brain, enabling computer programs to recognize patterns and to solve common problems in the fields of artificial intelligence, machine learning and deep learning.

Neural networks, also known as artificial neural networks (ANNs) or simulated neural networks (SNNs), are part of the machine learning function and form the basis of deep learning algorithms.

Artificial neural networks (ANNs) are composed of node layers that include an input layer, one or more hidden layers and an output layer. Each node (artificial neuron) connects to another and has an associated weight and threshold. If the output of a single node exceeds a certain threshold, that node is activated when sending data to the next network layer. Otherwise, no data are passed on to the next layer of the network.

How do neural networks work? Think of each individual node as a linear regression model composed of inputs, weights, variations (or thresholds) and outputs. The formula is thus as follows:

$$\overset{m}{\underset{i=1}{\sum }}w_{i}x_{i\;}+bias=w_{1}x_{1}+w_{2}x_{2\;}+w_{3}x_{3\;}+bias\;Output=f\left(x\right)=\{\begin{array}{c} 1\;if\;\sum w1x1+b\geq 0\\ 0\;if\;\sum w1x1+b<0 \end{array}$$

General formula describing the operation of a neuron:

y= f(s)

where in:

$$S=\overset{n}{\underset{i=o}{\sum }}x_{i}w_{i}$$

The activation function may take various forms depending on the specific model neuron. After determining the input layer, weights are assigned. Neural networks can be classified into different types and used for different purposes. The following list is not exhaustive; however, it is representative and presents the most common types of neural networks, with the oldest neural network being the perceptron, created by Frank Rosenblatt in 1958, with one neuron, and is the simplest form of neural network.

**Figure 3:**
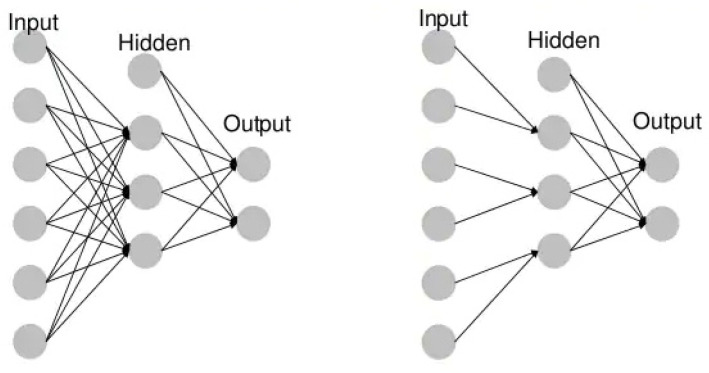
Example MLP vs. CNN.

- Unidirectional neural networks, i.e., multilayer perceptrons (MLP) (Figure 3), consist of an input layer, a hidden layer(s) and an output layer. While these neural networks are also commonly referred to as MLPs, keep in mind that they are actually sigmoidal neurons, not perceptrons, as most real-world problems are non-linear. Data are used to train these models. They form the basis of computer vision, natural language processing and other neural networks.
- Convolutional neural networks (CNNs) are similar to unidirectional networks but are typically used for image recognition, pattern recognition and/or computer vision. These networks use the principles of linear algebra, in particular, matrix multiplication, to identify patterns in an image.
- Recursive neural networks (RNNs) are distinguished based on feedback loops.

### 7.1. Neural Networks and Deep Learning

The terms “deep learning” and “neural networks” are often used interchangeably, which can be confusing. The word “deep” in “deep learning” only refers to layer depth in a neural network. A neural network that consists of more than three layers-including inputs and outputs-can be considered a deep learning algorithm. A neural network that has only two or three layers is just a basic neural network. The structure and use of deep nets has already been described in detail, which translates into the number of publications in the PubMed database. One of the newer publications, which is an interesting and modern comparison in the context of the discussed scoliosis, was presented by Chen et al. [65].

Between 2019 and 2021, the interest in artificial intelligence and methods such as deep learning and machine learning has seen an unimaginable increase; for example, they have begun to be used in the fight against COVID-19. The development of deep learning algorithms and methods also contribute to the development of other imaging methods and, consequently, diagnostics not directly related to COVID-19.

Traditional scoliosis screening methods are readily available but require referrals and radiographic exposures due to their low positive predictive value. The use of deep learning algorithms has the potential to reduce unnecessary referrals and, for example, scoliosis screening costs.

Publications directly related to the application of AI in scoliosis diagnosis that has appeared within two years are few thus far. The topic is evolving rapidly; however, the techniques are not yet used in standard diagnostics.

Yang et al. [66] presented an algorithm to identify cases with a curvature ≥ 20° and performed degree classification using uncovered back images with accuracy, sensitivity, specificity and positive predictive values (PPV) that are higher or comparable with those obtained by human experts. The use of algorithms can reduce the number of referrals, costs and time required for traditional scoliosis screening. Additionally, because deep learning algorithms (DLA) do not require radiation exposure, the method can be used as a periodic tool to monitor disease progression, thus avoiding excessive X-ray exposure. To our knowledge, this is the first large and complete study (including healthy control groups and different degrees of curvature) on intelligent scoliosis detection. The effectiveness of computer vision in scoliosis detection and classification has been demonstrated using uncovered back images.

Machine learning methods have already been used to detect spinal deformities using the torso surface defined by various techniques, including optical digitizing systems [67,68], orthogonal maps, surface topography techniques [69], laser scanners [70,71] and the Quantec system [72]. However, these methods still cannot be widely used due to the small scoliosis datasets, a lack of healthy control groups, the need for specialized equipment and the time-consuming nature of these methods. According to the authors, the above-mentioned methods, excluding X-rays, are perfectly sufficient. Limiting X-ray images is absolutely advisable and justified when specialists have alternative visualization methods.

### 7.2. Automatic Measurement Algorithm of Scoliosis Cobb Angle Based on Deep Learning

Zhang et al. [73] proposed a computer-aided Cobb angle measurement method based on Hough transform, which can automatically calculate the Cobb angle after manually selecting the region of interest (ROI) of the end circles and adjusting the brightness and contrast of the X-ray images. The Hough transform is based on the detection of regular shapes in computer vision. It is a special case of Radon transform known since 1917. The subject has developed relatively rapidly. In the paper by Samuvela et al. [74], an algorithm was presented to measure the Cobb angle. The algorithm was based on segmentation by applying a so-called mask. In another paper, Zhang [75] proposed an algorithm based on a deep neural network that can automatically estimate the slope of the spine after manually selecting the block of interest in the upper and lower vertebrae and can automatically measure the Cobb angle. Moreover, programs were also designed to measure the angle and improve the efficiency of radiologists [76,77]. As it turned out, the programs improved the efficiency of angle measurement; however, the upper and lower extremities of the vertebra had to be selected manually, which was time-consuming and subjective. This problem caused the development of more precise and stable methods. Image processing algorithms were improved, e.g., machine learning target detection algorithms [78] and algorithms for automatic image segmentation [79]. Thus, over several years, the methods have improved. The methods described above are related to the subjective experience of the clinician and contributed to the high measurement error of Cobb angles on scoliosis X-rays. Yongcheng et al. [80] proposed an automatic algorithm based on deep learning [81]. For spinal contour segmentation, they proposed DU-Net detection and segmentation network on spinal X-rays. The aggregated channel features in the detection algorithm are fed into the scoliosis image to detect the spine region. DU-Net is trained to segment the spinal contours. Therefore, the spine curve can be fitted to the spine contour, and the Cobb angle can be automatically measured using the tangent line of the spine curve. As a result, the Cobb angle automatic measurement method yields an average error of 2.9° compared with the orthopedist’s manual measurement.

Earlier methods of scoliosis evaluation based on segmentation consisting of filtering [73,82], active contouring [83] and physical models [84] localize the required vertebrae and calculate Cobb’s angle. These methods require the user to select circles, which is a limitation of these methods. As of 2021, no benchmarks, procedures or workflows have emerged to standardize the analysis performed and the selection of methods and algorithms.

In recent years, direct estimation methods [85,86,87], which aim to obtain relationships between medical images and clinical measurements directly without segmentation-based results, have achieved great success; they have been applied to measure scoliosis [85,86,87]. Unfortunately, these methods account for the basic relationship between AP and LAT X-rays but do not account for the unique features of AP and LAT projection images. Due to these limitations, Wang et al. [88] proposed an automated Cobb angle estimation method for scoliosis assessment using MVE-Net. They presented that MVE-Net effectively utilizes joint features and independent features in X-ray images from multiple perspectives. MVE-Net achieved high precision in Cobb angle estimation on both AP and LAT images in a large dataset of 526 X-ray images with different degrees of scoliosis. The computational method is also extendable to other clinical applications for high precision estimation.

Deep learning algorithms (DLAs) from CNNs, which have been applied to the detection of idiopathic scoliosis, were developed using 2D images [66] or Moiré topography [17,89,90]. Kokabu et al. [91] modified their system [92] to predict the Cobb angle even more accurately, which they successfully presented in their current publication.

## 8. Open Databases—Spine/Scoliosis Images

Despite a fair amount of publications on the topic of scoliosis as well as on the comparison of different methods, new papers continue to appear that are more and more accurate, structured and clear. One recent paper that appeared in early 2021 is truly recommendable and deals with the comparison of two current issues, scoliosis and machine learning in scoliosis diagnosis [93]. With the explosive growth of learning techniques and the topic of artificial intelligence in general, we decided to cover the databases that are essential for research towards artificial intelligence and scoliosis diagnosis. Several listed databases for medical diagnosis are free and are used by researchers around the world, including:

- PhysioNet (accessed on 1 December 2021)
- National Institutes of Health (NIH), e.g., https://clinicaltrials.gov/ct2/home, https://www.clinicaltrials.gov/ct2/show/NCT00448448?term=NCT00448448&rank=1 and https://www.niams.nih.gov/health-topics/scoliosis (accessed on 1 December 2021)
- Radiopaedia.org (accessed on 1 December 2021)
- ieee-dataport.org (accessed on 1 December 2021)
- https://stanfordmlgroup.github.io/competitions/mura/
- sethu.ac.in (accessed on 1 December 2021)
- boxdicom.com/samples.html (accessed on 1 December 2021)
- Biomedia (accessed on 1 December 2021)

When conducting this review, we noted the relative difficultly of acquiring data in the form of DICOM files of the spine in AP or LAT projection, CT and MRI. In the search process, we found “SpineWEb”, which is a database that contains several collections, and about 50 publications have already used their data [79,85,87,88,93,94,95,96,97,98,99,100,101,102,103,104,105,106,107,108,109,110,111,112,113,114,115,116,117,118,119,120,121,122,123,124,125]. A table (Table 1) focusing on the most important and current titles of publications created based on the “SpineWEb” database was prepared. It was divided into data quantity, algorithm, goals and results. Although vertebrae detection has been studied for years, reliably recognizing vertebrae from arbitrary spine MRI and TK images still remains a challenge.

## 9. Discussion

Forward bending tests, scoliometer measurements and individual Moiré topography are just some of the possibilities that unfortunately have various disadvantages that may have a direct impact on the diagnosis of patients. The disadvantages of these methods are considerable and include susceptibility to the subjectivity of the examiners and high time consumption, and we point out the need to perform radiography, which can have a direct negative impact on human health through the action of X-rays.

As presented in the literature [126,127], the possibilities of a quantitative assessment of spinal curvature have not yet been fully explored, thus leaving room for further improvement. Different papers/studies use different statistical methods and reproducibility of the chosen method, making it difficult to compare results. The so-called open databases, which can serve as a reference in some studies, thus become helpful. In this way, researchers have more methods of comparing the results and of gathering a large database that can be used in the next stage of research related to the application of artificial intelligence.

Artificial intelligence seems to be solving most problems connected to the repeatability of measurements or bias of researchers. Additionally, segmentation-based methods suffer from multiple error transmission because these methods are based on previous segmentation (manual or automatic) and then measure scoliosis based on this segmentation.

Typing “deep learning scoliosis” into the PubMed database, we found 14 results within the last 5 years. Although the method itself has been known for several decades, it has not yet found widespread use in the diagnosis of scoliosis. Soon, the interest in the use of artificial intelligence will increase.

## 10. Conclusions

Currently, the development of computational methods and their implementation in medicine contributes to improvements in health care procedures. The methods discussed often save time and, importantly, minimize human errors. Still, methods for assessing the curvature of the spine are developing dynamically, and many scientists are working on inventing a fully computerized method for quantitative assessment of curvature. At the moment, despite the advanced tools, we encounter a significant lack of repeatability of the results and the use of, for example, different statistical methods, which makes it difficult to compare the results. A good solution seems to be the creation of a database with exemplary reference values, which is missing at the moment.

## Figures and Tables

**Table 1:** Publications on spine image analysis based on “SpineWeb” (years 2019–2021).

Study/ Number of Data	Algorithms Applied	Objectives	Outcome Presentation
Liansheng W. [93] 707 spinal AP X-ray images	U-net	Top eight methods from twelve teams (including intuition, workflow, and implementation). Experimental results show that, overall, the best performing method achieved an asymmetric mean absolute percentage (SMAPE) of 21.7%.	Quantitative measurement of the spine.
Liyan L. [116] 895 axial spine MRI images from 143 patients	OSBP-Net, IPDC, and IICR	Applied to the output of the SFEs, taking into account that the activated regions in the feature maps of two paths should be theoretically different.	The prediction results, comparison with many other CADq models
Shen Z. [123] 450 MRI scans	Can-See is a two-step detection framework: (1) A hierarchical proposal network (HPN) to perceive the existence of the vertebrae. (2) A category-consistent self-calibration recognition (CSRN) network used to classify each vertebra and to refine their bounding boxes.	Category-consistent self-calibration recognition system (Can-See) used to accurately classify the labels and precisely predict the bounding boxes of all vertebrae with improved discriminative capabilities for vertebrae categories and self-awareness of false-positive detections.	Can-See achieves high performance (testing accuracy reaches 0.955) and outperforms other state-of-the-art methods.
Zhongyi H. [113] 253 clinical patients	Neural-symbolic learning (NSL) framework	Compares the semantic segmentation ability of a neural symbolic learning framework (NSL) with several state-of-the-art semantic segmentation networks (FCN, SegNet, DeepLabV3+, U-Net, Spine-GAN, GN-SGR, AGN-SGR, and AGN-DN).	NSL can directly generate radiologist-level diagnosis reports (using two steps) in spine radiology.
Dong Z. [118] 240 subjects	Sequential conditional reinforcement learning (SCRL). SCRL coordinates three major components (AMRL, Y-Net and FC-ResNet)	Propose a sequential conditional reinforcement learning network (SCRL) to tackle the simultaneous detection and segmentation of VBs from MR spine images.	SCRL achieves accurate detection and segmentation results, where on average, the detection IoU is 92.3%, segmentation dice is 92.6%, and classification mean accuracy is 96.4%.
Yanfei H. [117] 200 subjects	MMCL-Net: (1) The densely dilated ResNet, (2) The deep convolution level set module, (3) The instance feature merge module combines the global features extracted by DDRN and the local features obtained by segmentation	Novel end-to-end multi-task multi-structure correlation learning network (MMCL-Net) for the detection, segmentation and classification (normal, slight, marked and severe) of three types of spine structure: disc, vertebra and neural foramen simultaneously	MMCL-Net achieves high performance with a mAP of 0.9187, a classification accuracy of 90.67%, and a dice coefficient of 90.60%.
Liyan L. [116] 895 axial spine MRI images from 143 subjects	Dense enhancing network (DE-Net)	Dense enhancing network (DE-Net), which uses the dense enhancing blocks (DEBs) as its main body.	All deep learning models obtain very small prediction errors, and the proposed DE-Net with CSDPR acquires the smallest error among all methods.
Ranran Z. [115] 407 subjects	Multi-task relational learning network (MRLN)	A dilation convolution group is used to expand the receptive field, and LSTM (long short-term memory) to learn the prior knowledge of the order relationship between the vertebral bodies.	The accurate segmentation, localization and identification of vertebrae.
Jiawei H. [112] 320 axial lumbar MRIs	BS-ESNet	For the first time: (1) segmentation of the multiple paraspinal muscles and other major spinal components on axial lumbar MRIs simultaneously at both upper and lower spinal levels is achieved. (2) Boundary sensitive network provides a novel segment-then-detect workflow, which is robust to unclear organ boundaries and can further simplify multi-organ detection as an end-to-end trainable process; (3) Explicit saliency-aware network provides an elaborately designed architecture, which can utilize detection b-boxes to automatically correct and enhance segmentation features in an explicitly supervised manner and facilitates the adaptation of variable precise anatomical structures.	Proposal an explicit saliency-aware learning framework for segmentation of paraspinal muscles at varied spine levels.
Heyou Ch. [114] 292 MRI scans	A spatial graph convolutional network (GCN)	The proposed method is trained in an end-to-end.	Method achieves high performance (89.28 ± 5.21) of IDR and (85.37 ± 4.09%) of mIoU) from arbitrary input images.
Shen Z. [124] none	Adversarial recognition (FAR) network	Network to accurately perform spondylolisthesis grading by excellently detecting critical vertebrae without the need for locating landmarks.	Training accuracy: 0.9883 ± 0.0094, testing accuracy: 0.8933 ± 0.0276 for MRI images of different modalities, which can be attributed to the excellent critical vertebrae detection (detection mAP75 for training: 1 ± 0, for testing: 0.9636 ± 0.0180, and IoU (intersection-over-union) ≥ 0.9/0.8 for most detections with their corresponding ground truth in the training/testing dataset).
Liansheng W. [88] 526 X-rays	MVE-Net	Proposed multi-view extrapolation net (MVE-Net) that provides accurate automated scoliosis estimation in multi-view (both AP and LAT) X-rays.	Experimental results on 526 X-rays show 7.81 and 6.26 circular mean absolute error in AP and LAT angle estimation, which shows the MVE-Net provides an accurate Cobb angle estimation in multi-view X-rays
Shen Z. [123] none	Faster adversarial recognition (FAR)	Proposed faster adversarial recognition (FAR) network to accurately perform spondylolisthesis grading by excellently detecting critical vertebrae without the need for locating landmarks.	training accuracy: 0.9883 ± 0.0094, testing accuracy: 0.8933 ± 0.0276 for MRI images of different modalities, which can be attributed to the excellent critical vertebrae detection (detection mAP75 for training: 1 ± 0, for testing: 0.9636 ± 0.0180, and IoU (intersection-over-union) ≥ 0.9/0.8 for most detections with their corresponding ground truth in the training/testing dataset).
Shumao P. [111] MR images of 215 subjects	Cascade amplifier regression network (CARN)	Proposed novel cascade amplifier regression network (CARN) with manifold regularization including local structure-preserved manifold regularization (LSPMR) and adaptive local shape-constrained manifold regularization (ALSCMR) to achieve accurate direct automated multiple indices estimation.	Proposed approach achieves impressive performance with mean absolute errors of 1.22±1.04 mm and 1.24 ± 1.07 mm for the estimation of 30 lumbar spinal indices of the T1-weighted and T2-weighted spinal MR images, respectively.

## Data Availability

No new data were created or analyzed in this study. Data sharing is not applicable to this article.
